# Estimation of head motion in structural MRI and its impact on cortical morphometry

**DOI:** 10.3389/fnins.2026.1817743

**Published:** 2026-05-08

**Authors:** Charles Bricout, Samira Ebrahimi Kahou, Sylvain Bouix

**Affiliations:** 1 École de Technologie Supérieure, Montreal, QC, Canada; 2 Department of Electrical and Software Engineering, University of Calgary, Calgary, AB, Canada; 3 Canada Canadian Institute for Advanced Research (CIFAR) Artificial Intelligence (AI) Chair/Mila, Montreal, QC, Canada

**Keywords:** brain MRI, computer vision, cortical morphometry, deep learning, motion artifacts

## Abstract

Motion-related artifacts are inevitable in Magnetic Resonance Imaging (MRI) and can bias automated neuroanatomical metrics such as cortical thickness. These biases can interfere with statistical analysis which is a major concern as motion has been shown to be more prominent in certain populations such as children or individuals with ADHD. Manual review cannot objectively quantify motion in anatomical scans, and existing quantitative automated approaches often require specialized hardware or custom acquisition protocols. Here, we train a 3D convolutional neural network to estimate a summary motion metric in retrospective routine research scans by leveraging a large training dataset of synthetically motion-corrupted volumes. We validate our method with one held-out site from our training cohort and with 14 fully independent datasets, including one with manual ratings, achieving a Spearman Rank correlation of 0.71 vs. manual labels. We also tested the correlation of our predicted motion score with morphometric measurements known to be impacted by motion, achieving significant correlation on most datasets. Furthermore, our predicted motion correlates with subject age in line with prior studies. Our approach shows good generalization across scanner brands and protocols, enabling objective, scalable motion assessment in structural MRI studies without prospective motion correction. Finally, we provide empirical evidence that our motion estimator significantly improve model fitness when studying cortical thickness and volume. Our final model is made openly and freely available through “Agitation," a tool usable as a CLI, python package and integrated in Nipoppy and Boutiques. By providing reliable motion estimates, our method offers researchers a tool to assess and account for potential biases in cortical morphometric analyses.

## 1 Introduction

Motion artifacts are an inherent challenge in Magnetic Resonance Imaging (MRI). Zaitsev et al. (2015) explain that, as a single structural MRI acquisition lasts approximately 5 min, involuntary motion is inevitable. Even more problematic, this motion can vary widely in intensity, from undetectable to full corruption, and can manifest as blurring, ghosting, and fine concentric arcs. In fact, Andre et al. (2015) estimated that motion artifacts cost on average $115,000 per scanner per year. Multiple methods exist to reduce motion artifacts prospectively; Tisdall et al. (2011) proposed a technique called Volumetric Navigators (vNavs) relying on the acquisition of a low-resolution 3D volume throughout the acquisition sequence to compute and correct patient motion dynamically. Such methods, however, rely on a specific sequence and have a low temporal rate, rendering them unable to detect motion that occurs between navigator acquisitions. Zaitsev et al. (2015) estimates that this issue cannot be overcome solely through hardware improvements or a single methodological solution.

One of the multiple problematic aspects caused by motion is its impact on automatic measurement tools. Blumenthal et al. (2002) show that increasing levels of motion artifact, graded by manual labeling on a four-grade scale, are linked to a decrease in total gray matter volume estimation. Reuter et al. (2015) demonstrate this effect further by using several anatomical analysis packages, including FreeSurfer, a standard tool for automatic neuroanatomical computation Fischl, (2012). They show that increasing motion, estimated with vNavs, leads to decreased gray matter volume and cortical thickness estimations. Furthermore, Alexander-Bloch et al. (2016) find that there may be systematic effects of subject motion even on good quality volumes. They estimate the tendency of a subject to move by computing the average motion during an fMRI sequence and comparing it to estimates of cortical thickness, cortical gray matter volume, and mean curvature. They find that, as motion increases, thickness and volume decrease, whereas mean curvature increases. They also demonstrate that the effect of motion is not uniform throughout the brain by studying the thickness of the four separate lobes.

This impact on automatic measurement tools becomes more problematic as we explore differences in the tendency for motion between populations. Yuan et al. (2009) showed that age, sex, and task type all significantly influenced head motion in a cohort of 323 children aged 5 to 18 performing various language fMRI tasks. Pardoe et al. (2016) investigate if motion is related to diagnosis, age, and gender in a cohort of 2,141 subjects. They also estimate motion through fMRI and find a significantly increased tendency to move in subjects with Autism Spectrum Disorder (ASD), Attention Deficit Hyperactivity Disorder (ADHD), and schizophrenia; they also find similar tendencies in younger patients. Finally, they reinforce findings from Alexander-Bloch et al. (2016) by finding a significant relationship between fMRI-estimated motion and multiple anatomical metrics, such as gray matter volume and frontal, temporal, and parietal mean curvature, even when considering volumes that are manually assessed to be free of artifacts. These findings are very important as they may question results on those populations that do not take motion into account in statistical analyses. Andre et al. (2015) found that 19.8% of MRI examinations required at least one repeat sequence, i.e. re-acquisition of one or more sequences within the same scan session due to motion-degraded image quality.

Hence, there is a strong need for motion estimation techniques. First, Rosen et al. (2018) show a strong agreement between the Euler number, a cortical surface-based quality metric, and manual scoring of motion. They also show a significant relationship with the thickness of different regions of the brain. Although interesting, the Euler number is hardly interpretable, and manual scoring of motion is known to be noisy. Furthermore, their method is tested for generalizability on just one dataset, which might not be enough to guarantee robustness. On the other hand, Pollak et al. (2023a) use a depth camera mounted on an MRI to accurately quantify the motion of 500 patients. Custom software then approximates transformation matrices between successive head positions and computes the Root Mean Square (RMS) deviation, a metric proposed by Jenkinson (1999). They then train a Simple Fully Convolutional Network (SFCN) derived model to quantify motion, reaching an *R*^2^ of 0.433 on the test set, and also found a significant correlation with subjects' age. Unfortunately, the test set contains only 75 subjects and they did not use an external dataset to assess generalizability. This data labeling method, while accurate, demands specific materials and knowledge and can hardly be scaled for large data acquisition projects.

To address the manual labeling problem, several methods have turned to synthetic data generation. Mohebbian et al. (2021) use synthetic motion to corrupt images and label the created motion by computing the Frobenius norm of displacement and rotation applied. They then determine five bins of motion severity based on this norm and train an ensemble of Convolutional Neural Networks (CNNs), one for T1 and one for T2, to classify volumes. This ensemble model reaches an accuracy of 90.3% on the original dataset and 78.2% on an independent dataset. While the model is very accurate on synthetic data, it is not tested on real motion, and there is no proof of generalization to real data. It is also focused on 2D images, which might miss some artifacts. Finally, Sciarra et al. (2022) use a very similar approach, applying a ResNet on the Structural Similarity Index Measure (SSIM) between original data and corrupted data, grouped into 10 severity classes. They train on four datasets from different sites, reaching 89% accuracy on the test set. We identify similar limitations: they do not leave one dataset out for generalizability testing, models are only tested on synthetic artifacts, and the method uses 2D slices.

To address these limitations, we expand upon previous work (Bricout et al., 2025). In this paper, we (1) train a 3D SFCN on simulated motion artifacts quantified with RMS deviation, (2) compare our predicted score with manual labeling on real data, (3) extensively test our motion regressor on 15 real datasets, checking for previously reported correlations between motion and cortical morphometry measures, and (4) study the impact of adding our motion parameters on model quality in statistical analyses.

## 2 Method

### 2.1 Datasets

#### 2.1.1 Training data

We train our model using MRI from the Healthy Brain Network (HBN) dataset (Alexander et al., 2017). HBN is an initiative to acquire and share a biobank of data on 10,000 young New York area subjects, with ages ranging from 5 to 21. We use data acquired at three different sites. Data acquired at Rutgers University Brain Imaging Center (RUBIC) use a 3.0 T Siemens Tim Trio scanner, whereas data from City College of New York (CUNY) and CitiGroup Cornell Brain Imaging Center (CBIC) use a 3.0 T Siemens Prisma scanner; all use a 32-channel coil. The acquisition protocol for T1-weighted (T1w) volumes at all sites is derived from the Human Connectome Project (HCP) project (Marcus et al., 2013). Additionally, some of the volumes acquired at CBIC and CUNY also include T1w data with a protocol using vNavs, derived from the Adolescent Brain Cognitive Development (ABCD) study (Tisdall et al., 2011). This vNavs acquisition is designed to reduce the effects of motion; for this reason, we decide to use volumes from CBIC and CUNY to generate synthetic motion data. We use RUBIC to assess the relationship between motion and cortical thickness.

#### 2.1.2 Independent evaluation data

As our model is trained on synthetic artifacts only and on one dataset, we select a large number of datasets with varying protocols, hardware and populations intended for evaluating the model generalisabiliy.

**Movement-Related Artifacts (MR-ART)** is a dataset developed specifically to study the impact of motion on brain MRI (Nárai et al., 2022). It contains paired motion-free and motion-corrupted T1-weighted MRI scans from 148 healthy adults (ages 18-75). Structural volumes are acquired on a Siemens MAGNETOM Prisma 3T scanner with a 20-channel coil using a MPRAGE sequence with GRAPPA acceleration. For each participant, three scans were acquired with varying levels of motion:

- STAND: subjects have to stay still.
- HM1: subjects are instructed to nod their head five times when signaled.
- HM2: subjects are instructed to nod their head 10 times when signaled.

Then, two neuroradiologists with over 10 years of experience independently rated the clinical usability of all scans on a three-point scale:

- Good (1): diagnostically usable.
- Medium (2): partial artifacts, limited clinical utility.
- Bad (3): severe artifacts, unusable for diagnostics.

The raters were blind to the acquisition condition (STAND/HM1/HM2). To ensure consistency, they first harmonized their criteria on 100 independent scans and resolved ambiguities through consensus during the labeling process.

**Human Connectome Project Yound Adult (HCP-YA)** provides high-quality T1w brain MRI for 1,200 healthy adults (ages 22–35) (Van Essen et al., 2012). This study uses a modified Siemens Skyra (3T) scanner with a stronger gradient achieved by using an enhanced gradient coil designed for 7T scanners and a 64-channel coil. For the acquisition sequence, a version of MPRAGE with GRAPPA acceleration is used. This, combined with a thorough quality control process, ensures high-quality volumes.

**Human Connectome Project Early Psychosis (HCP-EP)** focused on studying the early stages of schizophrenia (Jacobs et al., 2025). The project recruited 303 participants aged 16-35, of which 299 were made available to us, we get a total of 383 high-quality T1-weighted brain MRI, as some participants were scanned multiple times. Participants were scanned across three different sites, each using a Siemens MAGNETOM Prisma 3T scanner with either a 32- or 64-channel coil and an MPRAGE sequence similar to HCP. It also provides a general quality score between one (worst) and four (best) for most volumes.

**Auxiliary test datasets from OpenNeuro:** we use OpenNeuro to find other suitable datasets to test our models. Our criteria are:

- Includes at least one subject between 5 and 21.
- Field strength should be three Tesla.
- Acquisition protocol should be similar to MPRAGE.
- No prospective motion correction techniques (e.g., vNavs).

The final dataset consists of 847 volumes gathered from 11 studies (Table 1). We have access to a variety of scanners; we are especially interested in ds000144 and ds003568 as they do not use Siemens hardware. ds005234 is also of interest as it uses a different Siemens scanner. While all studies use a sequence similar to MPRAGE, none employ identical parameters, which should also provide important variability for evaluating our method. We do not report these parameters as only partial information is available for each dataset, thus preventing direct comparison.

**Table 1:** Datasets retrieved from OpenNeuro.

Dataset	# Volumes	Age range	Population	Scanner
ds000115	99	11–30	Healthy/schizophrenic	Siemens Magnetom TrioTim
ds000144	45	6–10	Anxious children	GE Discovery MR750/Signa Excite
ds000256	24	5–15	Healthy	Siemens Magnetom TrioTim
ds001486	195	8–15	Healthy	Siemens Magnetom TrioTim
ds001748	62	10–35	Healthy	Siemens Magnetom TrioTim/Prisma
ds002424	79	8–12	Healthy/ADHD	Siemens Magnetom TrioTim
ds002862	71	8–10	Dyslexia	Siemens Magnetom Skyra
ds002886	56	8–15	Healthy	Siemens Magnetom TrioTim
ds003499	93	8–25	Healthy	Siemens Magnetom Prisma
ds003568	49	12–19	Healthy/depression	GE Discovery MR750
ds005234	74	6–13	Healthy/autism	Siemens Magnetom Verio

### 2.2 Training data generation

#### 2.2.1 Pre-processing

For pre-processing, we use Clinica's t1-linear pipeline (Routier et al., 2021). First, bias field correction is applied using the N4ITK method (Tustison et al., 2010). Next, an affine registration is performed using ANTs (Avants et al., 2014) to align each image to the MNI space with the ICBM 2009c nonlinear symmetric template (Fonov et al., 2011). This ensures data quality and that each brain is centered and approximately the same size.

Finally, we use Freesurfer v 7.4.1 (Athinoula A. Martinos Center for Biomedical Imaging) with the default configuration on all the preprocessed volumes to compute the thickness of 34 different structures and an aggregated mean cortical thickness for each subject (Fischl, 2012). This pipeline is applied to both training and independent evaluation data.

#### 2.2.2 Identifying “motion-free” data

As our training relies on simulated artifacts, we need to ensure that the volumes used for the synthetic generation process are as motion-free as possible. This helps reduce noise in our training data by making our synthetic labels as accurate as possible. We created a simple web-based tool to review the volumes from each site with a pass or fail rating. A volume would fail if any sign of motion could be seen on one of the three slices sampled from the volume.

We later improved this rating system with six possible labels: “Clean,” “Barely Noticeable,” “Noticeable,” “Strong,” “Unusable,” and “Corrupted,” to allow filtering on a finer level if the quantity of “Clean” and “Barely Noticeable” data were insufficient. We rate only the CUNY dataset using the six-point Quality Control (QC) scale and retain only volumes rated as “Clean” or “Barely Noticeable” for the next step. In total, we retain only 449 volumes out of the 4,079 available.

All volumes were reviewed by Rater One (CB). To assess reliability, we asked two additional independent raters to grade the same 50 randomly selected volumes. Below, we provide information about motion artifact effects and our grading scale:

1. “Clean”: no doubt about data quality.
2. “Barely noticeable”: unsure but no clear effects.
3. “Noticeable”: clear lines or blurring, noisy white matter.
4. “Strong”: strong lines and blurring, unclear delimitation between gray and white matter.
5. “Unusable”: hard to distinguish any information.
6. “Corrupted”: corruption unrelated to motion (truncation, metal artifacts, etc.).

Representative examples for each label are also provided (Supplementary Figure S2). Inter-rater reliability is assessed using the Intraclass Correlation Coefficient (ICC) (Shrout and Fleiss, 1979). We employ a two-way random-effects, average-measure, absolute-agreement model (ICC(2,k)). We use the same metric for intra-rater reliability by asking Rater One to grade the 50 selected samples 6 months after the initial grading. Overall, we observe significant inter- and intra-rater reliability, confirming the reproducibility of our volume selection method [Intra-rater ICC(2,k) = 0.903, *p* < 1*e*−20, 95*%CI* = [0.8, 0.95], Inter-rater ICC = 0.883, *p* < 1*e*−10, 95*%CI* = [0.78, 0.94]].

#### 2.2.3 Synthetic motion generation

To create our synthetic data, we apply random synthetic motion using TorchIO (Pérez-García et al., 2021). This transformation samples *N* transformation matrices representing subject motion, constrained by maximum translation and rotation parameters, and concatenates their k-space into a final, corrupted k-space (Shaw et al., 2019). Using the successive transformation matrices, we can quantify the motion using the RMS deviation (Jenkinson, 1999). It is calculated as follows: let *T*_1_ and *T*_2_ be two transformation matrices, and let $x_{c}\in {\mathbb{R}}^{3}$ be the center of the MRI volume. We define *A* and *t* as:


$$\begin{array}{l} M=T_{2}T_{1}^{-1}-I\\ =\left[\begin{array}{cc} A & t\\ 000 & 0 \end{array}\right]. \end{array}$$

Given an estimation of the distance from face to head center *R*_*c*_ = 82.5mm (Pollak et al., 2023b), the RMS deviation is then given by:


$$\begin{array}{l} E_{RMS}=\sqrt{\frac{1}{5}R_{c}^{2}\text{Trace}\left(A^{⊤}A\right)+{\left(t+Ax_{c}\right)}^{⊤}\left(t+Ax_{c}\right)}. \end{array}$$

To ensure a uniform distribution of motion data, we sample an expected motion magnitude from a uniform distribution, $e_{\text{motion}}\sim U\left(0.01,4.0\right)$. Using the expected motion, we sample reasonable restrictions for maximum rotation and translation from the following distributions: $c_{\text{translation}}\sim U\left(0,\max\left(e_{\text{motion}},1\right)\right)$ and $c_{\text{rotation}}\sim U\left(0,\max\left(2\times e_{\text{motion}},1\right)\right)$. These values are found empirically. With our constraints, we can finally sample a full transformation and compute the RMS deviation. As each transform is randomly generated to roughly match a predetermined motion score, we defined a tolerance parameter of 0.02 to control whether we need to sample a new transformation; we repeat this process until we obtain a transformation that satisfies the tolerance.

To augment the anatomical variety of our dataset, we apply random sagittal flipping (random left-right mirroring about the mid-sagittal plane, *p* = 0.5) and elastic deformation before generating the synthetic motion. We generate 300 synthetic volumes for each original clean volume, resulting in 137,700 synthetic volumes that we split for train, validation, and test in a 60-20-20 split. To study the effect of synthetic motion on cortical morphometry, we also generate 50 samples for 20 random subjects of the selected volumes with elastic transformation and random flip disabled, resulting in 1,020 volumes when accounting for the original volumes.

### 2.3 Neural network architecture and training strategy

#### 2.3.1 Model architecture

**Figure 1:**
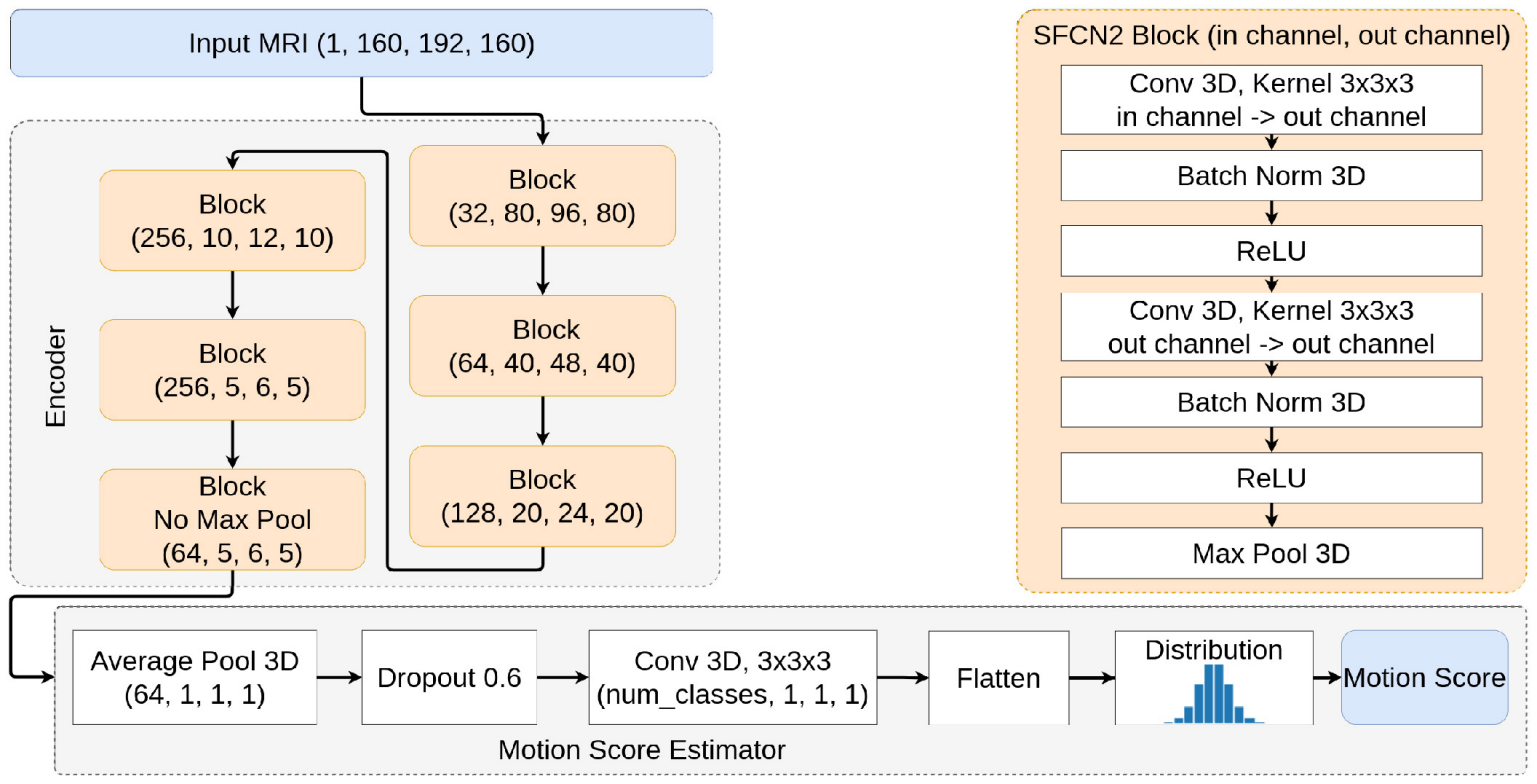
Description of the SFCN2 architecture.

Expanding on our previous study on synthetic motion prediction, we use the SFCN (Figure 1) as our baseline architecture (Bricout et al., 2025). SFCN is a lightweight 3D CNN architecture first proposed for brain age prediction (Peng et al., 2021) and then used for head motion estimation (Pollak et al., 2023a).

Instead of directly regressing the motion score, we define a range of possible motion [–0.5,4.5] that we discretize in *N*_bins_ = 50. Then, the network learns to predict a distribution over these bins, representing the probability of the noise magnitude. Finally, we train the CNN by minimizing the Kullback-Leibler (KL) divergence between the predicted distribution and a normal distribution centered on the true motion score.

Our model's encoder consists of six blocks, with five blocks performing downsampling and a sixth block adding more non-linearity and parameters to our network without changing the shape of our data. We also use the proposed channel configuration for each layer: [32, 64, 128, 256, 256, 64, 40]. For motion inference, we compute a weighted average of each motion bin:


$$\text{motion score}=\overset{N_{\text{bins}}}{\underset{i}{\sum }}x_{i}\cdot {\text{motion}}_{i}$$

where *x*_*i*_ is the predicted probability for the *i*-th bin, and motion_*i*_ is the center value of the *i*-th bin.

Our model is a slightly heavier variation of SFCN, using two convolutions per block instead of one. This architecture is more expressive, which might help capture subtlety while still being light enough for efficient training. We refer to this network as SFCN2.

#### 2.3.2 Training

We train our models using the AdamW optimizer with a learning rate of 0.001 and a weight decay of 0.1, both selected empirically. We chose high values for these two hyperparameters to avoid overfitting on synthetic data. We use a dropout rate of 0.6, also found empirically, and a batch size of 10, which is the maximum we can fit on one GPU. Models are trained using Digital Research Alliance of Canada's Narval computing cluster on four Nvidia A100 GPUs, using 20 CPU cores and 100 GB of RAM. The training procedure completes in approximately 32 h for all models. We train for 80,000 steps and select the model with the lowest Jensen-Shannon divergence between the validation set label distribution and the distribution of all our predicted labels. We also report metrics such as *R*^2^ and Root Mean Square Error (RMSE), but we decide to use the Jensen-Shannon divergence as the selection criterion, as we notice a tendency for predictions to concentrate at the extreme ends of the motion range. The Jensen-Shannon divergence helps us select the model that best represents the overall motion distribution.

##### 2.3.2.1 Training time augmentation

As different datasets can have widely different contrasts and artifact levels, we use TorchIO to simulate Gaussian noise, Gaussian blur, and Gamma corrections (Pérez-García et al., 2021). As we aim to make our model as robust as possible to different datasets, we strive for broad contrast simulation and also use MONAI's random histogram shift (Cardoso et al., 2022). We also apply a random flip along the sagittal plane.

For a given volume, we separate our augmentation pipeline into two stages:

- Base augmentation: we apply a flip with a probability of 0.5 and either add random Gaussian noise or apply smoothing with a probability of 0.8.
- Contrast augmentation: we apply either random gamma changes or a histogram shift on every volume.

**Figure 2:**
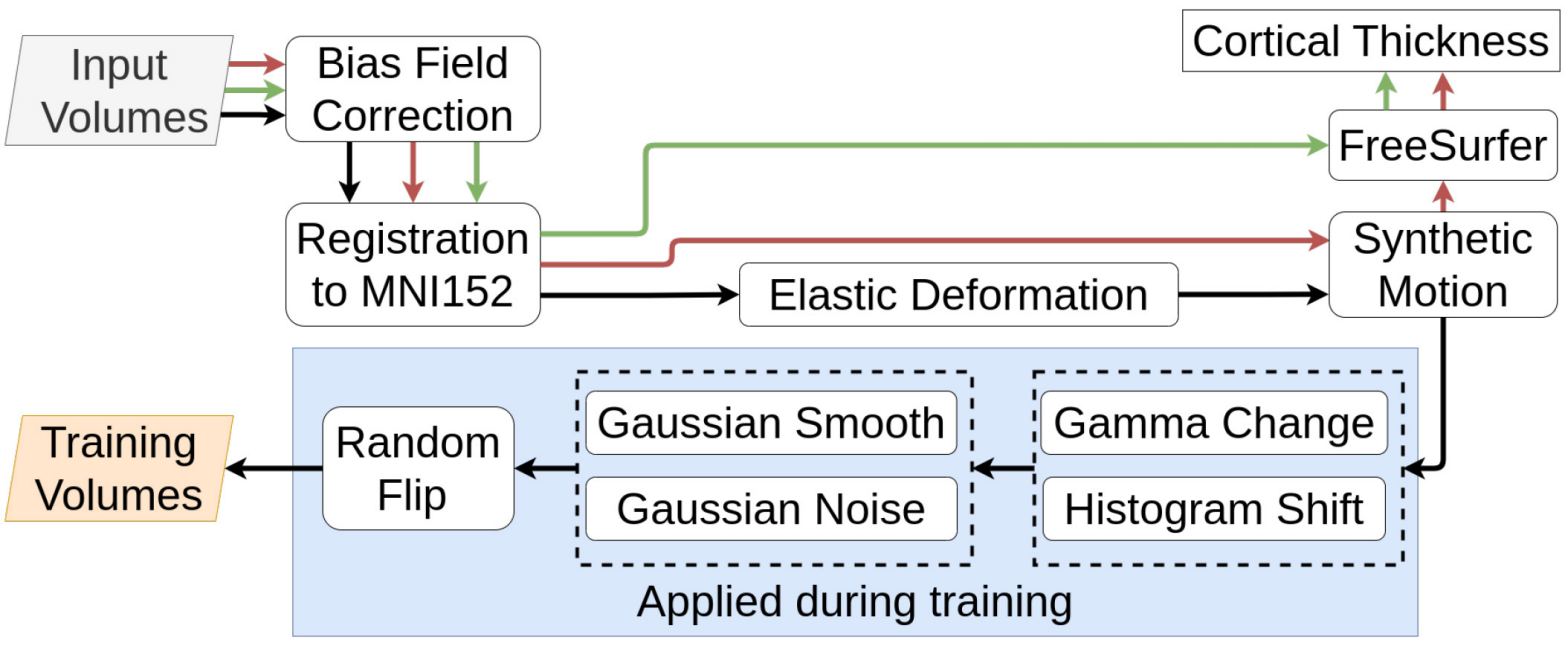
Data processing flow. **Black arrows** represents training data, **red arrows** represents data used to assess synthetic motion effect, and **green arrows** represents test data from real datasets.

For each stage, we randomly apply one of the proposed transformations to avoid augmenting a volume to the point of corruption. Figure 2 illustrates the full data processing pipeline for both inference and training.

Finally, to reduce the computational load inherent to 3D CNNs, we crop our volume to a ROI of (160, 192, 160) and, to ensure consistency between volumes, we scale voxel intensities to the (0,1) range using min-max scaling.

### 2.4 Evaluation of model performance

Our first set of experiments focus on evaluating the trained model by comparing its prediction with ground truth synthetic labels and a human rated motion score.

#### 2.4.1 Performance on synthetic data

We first assess the performance of our method in the synthetic test set. This dataset is produced by applying 300 different motion corruption to each data point on 20% of the HBN Site-RUBIC dataset, for a total of 27, 540 volumes. Our goal is to confirm that the model correctly learned the synthetic labels. For this, we compute the *R*^2^ correlation between our predicted score and the true synthetic labels. We also visually analyze the quality of the predictions with a scatter plot.

#### 2.4.2 Comparison with real motion

We then test the model's ability to directly quantify motion on real artifacts using the three-level motion scores provided with the MR-ART dataset (see Section 2.1.2). We compute the Spearman rank correlation between our continuous predictions and MR-ART's visual assessment scores. This experiment evaluates the level of agreement between human rated motion through visual inspection and the predicted motion score from our model in a dataset with real motion.

### 2.5 Confirmation of known relationships between motion, thickness and age

As stated previously, motion is known to be correlated with cortical thickness (Reuter et al., 2015; Alexander-Bloch et al., 2016; Madan, 2018) and certain phenotypic attributes, such as age (Pardoe et al., 2016). We therefore test whether our motion prediction score is correlated with cortical thickness (specifically the mean left cortical thickness) and age. These experiments are performed on multiple datasets with different acquisition settings, thereby evaluating both the accuracy and robustness of the model.

#### 2.5.1 Biases in synthetic motion

We start by verifying that synthetic motion does introduce these biases by comparing the mean left hemisphere thickness with the synthetic motion label. This experiment assesses the adequacy of the synthetic motion as a proxy for real motion.

First, we plot the cortical thickness of the left hemisphere as a function of the motion score. We also compute the percent difference between the original thickness and the thickness of the corrupted scan by subtracting the motion-corrupted scan thickness from the original motion-free scan thickness.


$$Loss\left(\text{original},\text{synthetic}\right)=\frac{Thickness_{\text{synthetic}}-Thickness_{\text{original}}}{Thickness_{\text{original}}}$$

×100

Second, using the statsmodels library, we fit a Generalized Linear Model (GLM) for the following relationship:


$$\begin{array}{l} \text{Thickness}=\beta_{1}\cdot \text{age}+\beta_{2}\cdot \text{sex}+\beta_{3}\cdot \text{motion}+c \end{array}$$ (1)

where “Thickness” is the mean cortical thickness of the left hemisphere. We used the default parameters: Gaussian family with the identity link function. Our goal is to determine whether the motion variable significantly impacts thickness in a statistical model. We define that a correlation is significant if *p* < 0.05.

#### 2.5.2 Impact of estimated motion on cortical thickness in real data

To study the correlation between our motion score and cortical thickness, we start by qualitatively visualizing the relationship between motion and cortical thickness for HBN site RUBIC, MR-ART, HCP-EP and HCP-YA. We also plot the distribution of motion predictions for each dataset.

We then assess these correlations from a quantitative standpoint by fitting the statistical model specified by Equation 1 for each dataset identified from OpenNeuro and the previously mentioned datasets. We report *p*-values and coefficients associated with the motion parameter. Moreover, as previous studies have shown that some structures are more impacted by motion than others, we also fit a model for each of the 34 structures' thicknesses reported by FreeSurfer. As we test 15 different datasets, we obtain 15 × 35 = 525 models; hence, we apply Benjamini-Hochberg's False Discovery Rate (FDR) correction (Benjamini and Hochberg, 1995) to the full set of *p*-values to correct for accidental significance.

#### 2.5.3 Correlation between estimated motion and age

As stated previously, multiple studies observe higher motion in younger and older subjects compare to middle aged individuals. To further validate our models, we test for the same type of relationship by fitting a GLM for the relationship:


$$\begin{array}{l} \text{motion}\sim \beta_{1}\cdot \text{age} \end{array}$$

We test this relationship in HBN's RUBIC site, MR-ART, and on the aggregation of all OpenNeuro data. We exclude HCP-YA and HCP-EP as their age range was between 18 and 36 years old, which is not reported to be strongly correlated with motion (Pardoe et al., 2016).

### 2.6 Exploration of the impact of estimated motion on cortical morphometry

After validating our motion predictor with multiple experiments on a variety of datasets, we will use it to assess the impact of motion on different morphological measurement of cortical substructures and on statistical modeling.

#### 2.6.1 Frequency analysis on thickness, area, and volume

Using the same procedure described in Section 2.5.2, we study the impact of estimated motion on the thickness, area and volume of each structure defined by the Desikan-Killiany parcellation provided by FreeSurfer. We aggregate the results from the 15 datasets and compute how often each region is significantly correlated to motion across those 15 datasets. We want to determine if some regions are more frequently impacted by motion and if there are measurements (thickness, volume or area) that are more sensitive to motion.

#### 2.6.2 Evaluation of statistical model quality with and without motion estimation

One of the main hypothesis of our study is that one should incorporate a motion score in statistical modeling to improve robustness by correcting for motion biases. To test the capacity of our estimator to improve statistical modeling, we compare the Akaike Information Criterion (AIC) between two models:


$$\begin{array}{l} \text{Thickness}=\beta_{1}\cdot \text{age}+\beta_{2}\cdot \text{sex}+\beta_{3}\cdot \text{motion}+c \end{array}$$

and


$$\begin{array}{l} \text{Thickness}=\gamma_{1}\cdot \text{age}+\gamma_{2}\cdot \text{sex}+c \end{array}$$

The AIC is defined by Akaike (1998) as:


$$\begin{array}{l} AIC=2k-2\ln\left(L\right) \end{array}$$

where *k* is the number of parameters in the model and *L* is the maximum likelihood of the model. The AIC represents the trade-off between the complexity and the likelihood of the model, which allows us to compare models with different parameters. In order to quantify the impact of the motion variable on the fitness of the model, we compute the difference between the AIC of both models:


$$\begin{array}{l} \Delta_{AIC}=AIC_{base}-AIC_{motion} \end{array}$$

Extending the thresholds provided by Burnham and Anderson (2004) to negative values, we give this interpretation:

- Δ_*AIC*_ < −10 : considerable evidence that the model **without motion** is better.
−7 ≤ Δ_*AIC*_ < −4 : moderate to high evidence that the model **without motion** is better.
−2 ≤ Δ_*AIC*_ < 2 : negligible difference.
- 4 ≤ Δ_*AIC*_ < 7 : moderate to high evidence that the model **with motion** is better.
- Δ_*AIC*_>10 : considerable evidence that the model **with motion** is better.

We compare the *AIC* on the mean cortical thickness for both hemisphere, and we also compute the Δ_*AIC*_ for all APARC regions. We also compute these values for area and volume (Supplementary Section 2.3).

## 3 Results

Our experiments aim to (1) assess our model's performance for motion prediction on both synthetic and real data; (2) validate this estimated motion parameter by evaluating previously reported associations between motion and cortical morphometry; (3) study the impact of motion on other structures and measurements; and (4) evaluate the impact of our motion estimation tool when fitting models in neuroscience.

### 3.1 Model performance

#### 3.1.1 Performance on synthetic data

**Figure 3:**
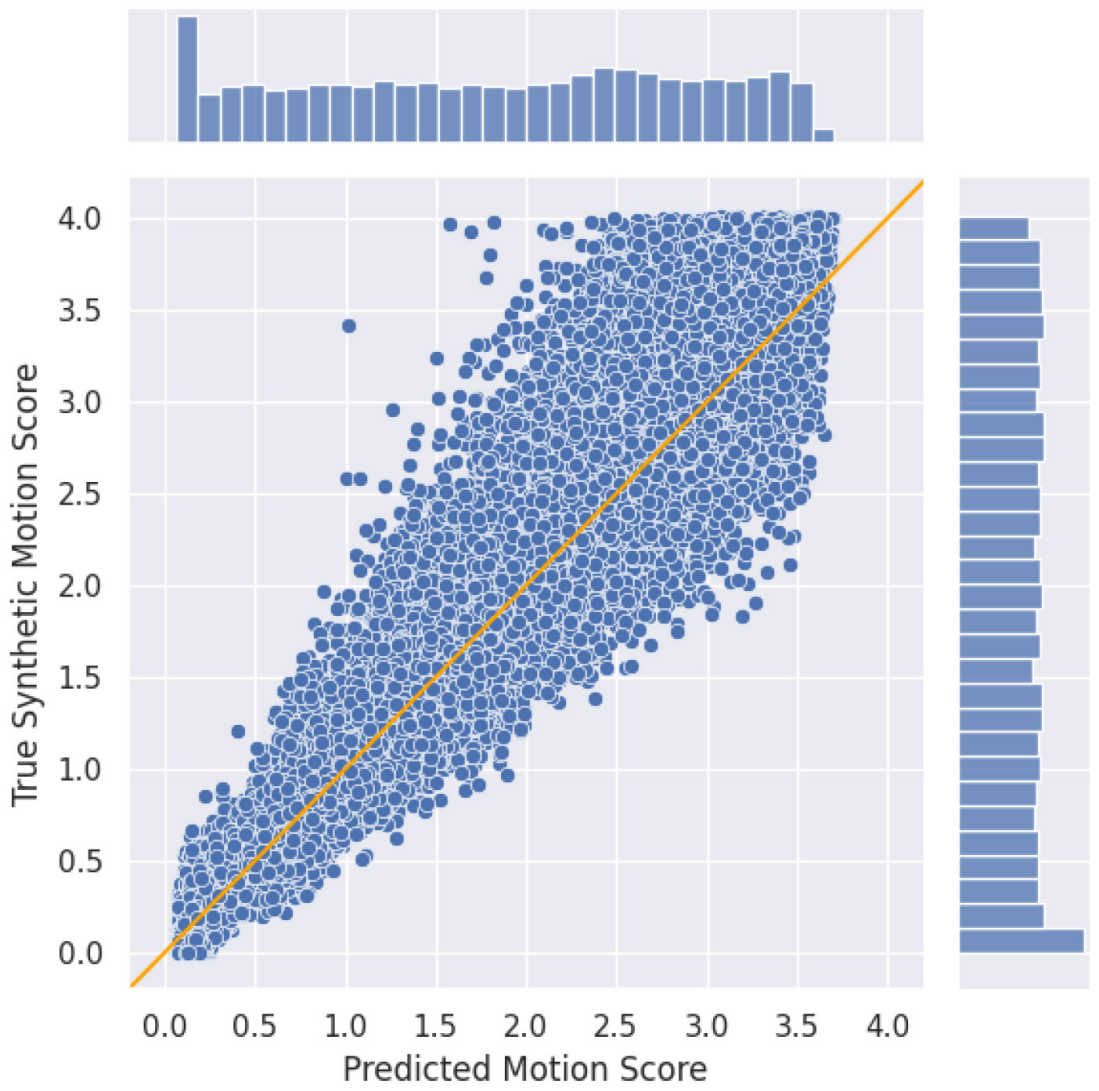
Prediction on the synthetic test set for our model compared to ground truth. The orange line represents *y* = *x*: the perfect prediction.

In the prediction plot (Figure 3), we notice that our predicted motion values are concentrated near zero, with no values exceeding approximately 3.5. We also observe greater deviation from the *y* = *x* line as the motion increases. This effect can be explained by the nature of our task: we quantify the actual simulated motion rather than the effect of motion on the volume. A single motion score can correspond to different expressions of artifacts, which become harder to distinguish as the motion level increases.

#### 3.1.2 Comparison with real motion

Our model achieves a high Spearman rank correlation of 0.71 between estimated motion and the scores from human experts in the MR-ART dataset. This result demonstrates our model's capability for real motion prediction without fine-tuning on independent data.

#### 3.1.3 Ablation study

To assess the benefits of our approach, we perform an ablation study starting from the base SFCN to our two-convolution SFCN with both augmentation types (Table 2). The results indicate that the SFCN2 without any augmentation outperforms the other variations on the synthetic test set. We explain these results by a discrepancy between the train/validation and test sets when augmentation is applied. Indeed, our augmented networks are exposed to a different domain that does not match the test set domain, which could explain the underperformance compared to a network specifically trained on this domain. We understand that the added expressiveness given to SFCN2 is beneficial for this task.

**Table 2:** Results of variations of SFCN on synthetic test set and MR-ART dataset.

Conv.	Augmentation		Synthetic test set		Spearman rank
	Base	Contrast	*R*^2^ score	RMSE	MR-ART
1	✗	✗	0.917	0.339	–0.10
2	✗	✗	**0.940**	**0.287**	0.11
2	✓	✗	0.921	0.330	0.66
2	✓	✓	0.898	0.375	**0.71**

SFCN2 without any augmentation performs better on synthetic data, but SFCN2 using all augmentation strategies has a stronger correlation with real motion scores. Best scores are highlighted in bold.

**Figure 4:**
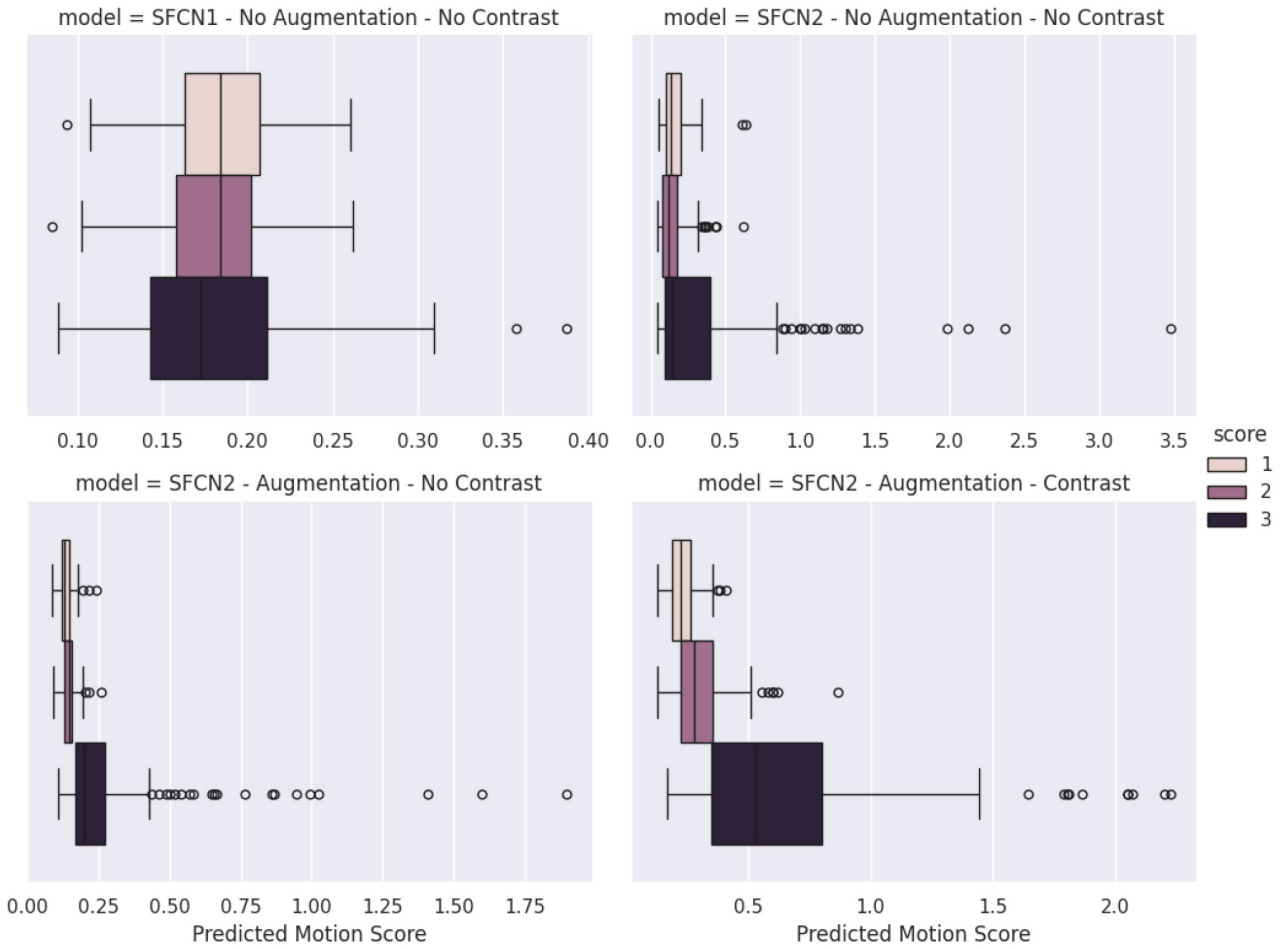
Predicted motion score distribution for each MR-ART motion label. The original SFCN (**top left**) shows no clear separation between motion classes. In contrast, the SFCN2 model with augmentations (**bottom left and bottom right**) demonstrates a clear ranking of motion scores, particularly when using contrast augmentation, indicating improved class separability.

On the MR-ART manual labels, the complete model outperforms the other variations by a large margin. This confirms that our model is more robust in real scenarios, even if its performance on synthetic data is lower. It is also interesting to note that the introduction of Gaussian noise and smoothing (base augmentation) greatly improves our motion regression capability to accurately rank the MR-ART labels. These two augmentation steps appear to make our model more robust to real and unseen data. Visually, we also observe that our model's predictions follow the MR-ART grading (Figure 4). A label of 1 corresponds to volumes with nearly no artifacts, whereas a label of 3 encompasses all magnitudes of severe artifacts. We can see this difference in range in the interquartile distances of our boxplots. Finally, we also visualize the uncertainty of intermediate classes, as the label 2 grade is not as distinctly separated.

### 3.2 Known relationships between motion, Thickness, and age

#### 3.2.1 Relationship between thickness and ground-truth synthetic motion

**Figure 5:**
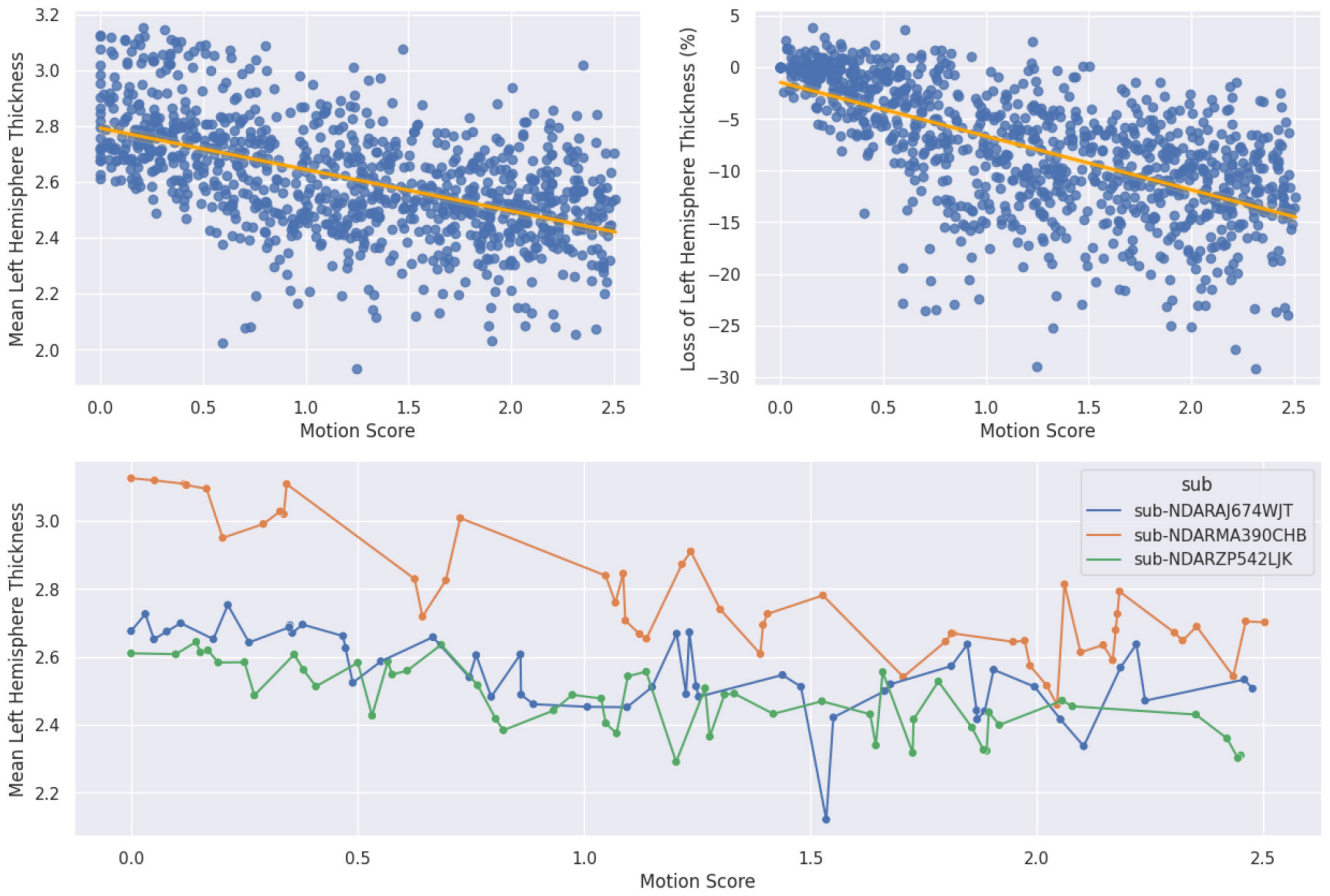
First row linear regressions of order one between motion score and left hemisphere thickness **(left)** and percent loss in left hemisphere thickness **(right)**. Second row: evolution of cortical thickness as we generate an increasing amount of motion on the same volumes.

We visualize the downward trend in Figure 5 and we obtain a pseudo-*R*^2^ of 0.583, as well as a very low *p*-value, indicating a highly significant correlation between the ground truth motion score from synthetic data and FreeSurfer's cortical thickness (Table 3). As this negative correlation is known to occur with real data, finding the same kind of relationship validates the assumption that training on synthetic artifacts may help estimate this bias with real volumes.

**Table 3:** GLM regression results.

	Coefficient (β_*i*_)	Standard error	*p* > |*z*|	[0.025 0.975]	
Intercept	3.1017	0.027	0.000	3.049	3.155
Age	–0.0229	0.002	0.000	–0.026	–0.020
Sex	0.0345	0.011	0.001	0.013	0.056
Motion score	–0.1480	0.006	0.000	–0.161	–0.135

We obtain a pseudo-*R*^2^ of 0.583 and a *p*-value of 2*e*^−116^ for the motion parameter.

#### 3.2.2 Correlation between estimated motion and cortical thickness in real data

**Figure 6:**
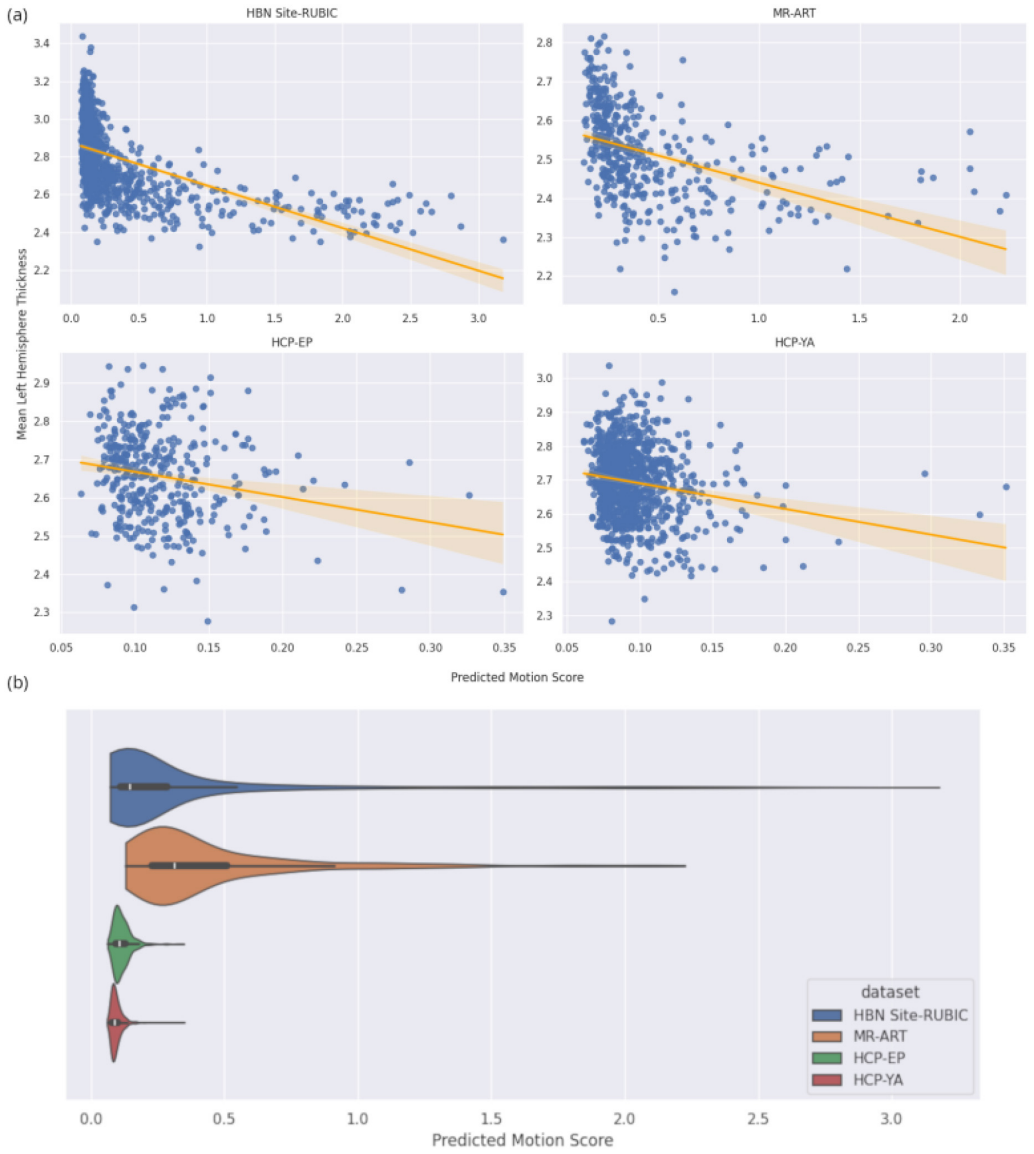
**(a)** Regression between mean left hemisphere cortical thickness and our model's predicted motion score. There is a clear negative correlation for HBN and MRART (top left and top right). In contrast, the correlation for HCPEP and HCPYA (bottom left and top bottom) is weaker. **(b)** Predicted motion score distribution for each dataset.

While we can see a clear downward trend in HBN and MR-ART, we notice that plots from HCP show more uncertainty toward larger motion scores (Figure 6). We also notice a stark difference in motion distribution. This may be due to the fact that HBN subjects are children to young adults—a population more prone to movement—while MR-ART's MRI scans are motion-corrupted by asking participants to move their head in the scanner. In contrast, HCP's strict quality standards and more mature cohorts should exhibit weaker motion artifacts.

We obtain significant relationships in 12 of the 15 datasets, which further indicates strong generalizability of our model (Table 4). Furthermore, analyzing the motion coefficients for significant datasets, we find an average value of –0.208 ± 0.088, which agrees with the negative relationship observed in the literature and our synthetic data study. Moreover, we obtain a strong correlation with ds000144 and ds005234, showing robustness to different hardware.

**Table 4:** Results of fitting Equation 1 for each dataset.

Dataset	Motion's impact on mean thickness			Percentage of
	Coefficient	*p*-value	FDR corrected *p*-value	Significant structures
HBN Site-RUBIC	–0.246	1.55e–143	**2.04e–141**	97.14%
HCP-YA	–0.651	2.73e–07	**1.75e–06**	77.14%
HCPEP	–0.904	1.21e–03	**3.64e–03**	51.43%
MR-ART	–0.124	1.19e–21	**1.56e–20**	85.71%
ds000115	–0.232	1.98e–04	**7.42e–04**	54.29%
ds000144	–0.301	3.72e–07	**2.32e–06**	51.43%
ds000256	–0.173	8.56e–05	**3.60e–04**	54.29%
ds001486	–0.100	3.42e–07	**2.16e–06**	77.14%
ds001748	–0.068	2.57e–01	3.47e–01	17.14%
ds002424	–0.213	5.46e–07	**3.22e–06**	74.29%
ds002862	–0.229	3.64e–03	**9.00e–03**	40.00%
ds002886	–0.082	1.66e–02	**3.43e–02**	17.14%
ds003499	0.075	6.80e–01	7.46e–01	2.86%
ds003568	–0.366	1.73e–01	2.49e–01	11.43%
ds005234	–0.332	2.63e–11	**2.51e–10**	68.57%

*p*-values are adjusted with false discovery rate correction; bold *p*-values indicate < 0.05.

With respect to our analysis of individual structures, we find that at least one structure is impacted by motion in every dataset. Given a conservative correction of *p*-values, this shows that our model detects a bias in every dataset.

#### 3.2.3 Correlation between estimated motion and age

We find significant relationships for all datasets (Table 5). It is worth noting that we obtain a negative coefficient for OpenNeuro and HBN Site-RUBIC, which have age ranges of 5–35 and 5–21, respectively, whereas we find a positive coefficient for MR-ART, which has subjects between 18 and 75. These findings are in line with the literature with motion being more prevalent in younger children and older adults compare to young and middle aged adults.

**Table 5:** Relationship between motion predicted by our best model and subject age.

Dataset	Age			
	Coefficient	*p*-value	FDR Corrected *p*-value	Range
HBN Site-RUBIC	–0.022	9.78e–08	**2.93e–07**	5–21
MR-ART	0.004	1.86e–03	**1.86e–03**	18–75
All OpenNeuro data	–0.010	1.14e–06	**1.71e–06**	5–35

Bold *p*-values indicate < 0.05.

### 3.3 Impact of estimated motion on cortical morphometry

#### 3.3.1 Frequency analysis on thickness, area, and volume

**Figure 7:**
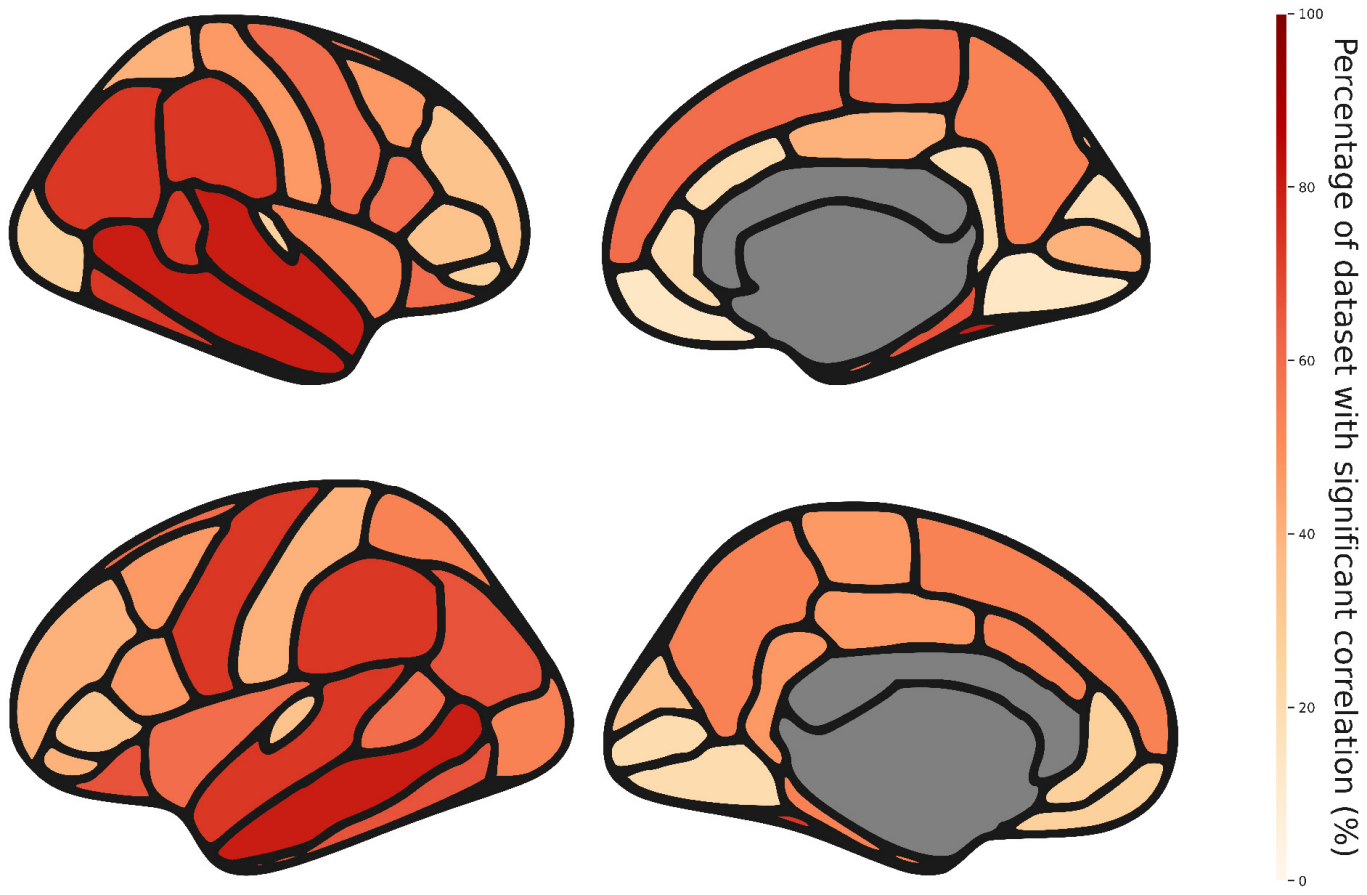
Percentage of dataset with a significant correlation between thickness and motion for each structure.

For thickness, we report that 35 out of 68 regions are affected in more than 50% of the datasets (Figure 7). We can also observe that some regions like middle-temporal and superior-temporal are often affected by motion, whereas the Lingual and medial-orbito-frontal regions show correlation with motion in only two out 15 datasets (Supplementary Table S1). We might want to be especially cautious when studying these highly impacted regions. Additional tables with detailed measures are available in the Supplementary material.

With regards to other volume and area, we observe that volume, while still often affected by motion, is less vulnerable to motion than thickness (see Supplementary Figure S4). Area is least often affected by motion overall, no region reported a frequency of correlation over 50% (see Supplementary Figure S3). For every measurement, while there are some variations between the two hemisphere, no notable pattern can be identified.

#### 3.3.2 Impact on statistical model quality

We see that a statistical model incorporating motion is considerably better for nine of the datasets (see Table 6). We also notice that the model with motion is detrimental in only three datasets, and, as Δ_*AIC*_ > −2 for all datasets, there is no evidence that introducing the motion can have a noticeable negative impact. Expectedly, these three datasets showed no significant correlation to motion (see Section 3.2.2).

**Table 6:** AIC comparison on the impact of introducing a motion parameter when modeling the mean cortical thickness for each hemisphere.

Dataset	Hemisphere	Δ_*AIC*_	*AIC*_*base*_	*AIC*_*motion*_
HBN Site-RUBIC	Left	503.40	–522.93	–1,026.33
HBN Site-RUBIC	Right	503.40	–522.93	–1,026.33
MR-ART	Right	81.63	–719.67	–801.29
HBN Site-RUBIC MR-ART	Left	81.63	–719.67	–801.29
ds005234	Left	33.96	–82.00	–115.96
ds005234	Right	33.96	–82.00	–115.96
HCP-YA	Right	24.24	–1,803.28	–1,827.52
HCP-YA	Left	24.24	–1,803.28	–1,827.52
ds001486	Left	22.88	–245.68	–268.56
ds001486	Right	22.88	–245.68	–268.56
ds002424	Left	20.80	–103.35	–124.15
ds002424	Right	20.80	–103.35	–124.15
ds000144	Right	19.99	–63.19	–83.18
ds000144	Left	19.99	–63.19	–83.18
ds000256	Right	11.72	–20.40	–32.12
ds000256	Left	11.72	–20.40	–32.12
ds000115	Left	11.47	–156.60	–168.07
ds000115	Right	11.47	-156.60	–168.07
HCPEP	Left	8.40	–288.70	–297.10
HCPEP	Right	8.40	–288.70	-297.10
ds002862	Left	6.44	–100.68	–107.12
ds002862	Right	6.44	–100.68	–107.12
ds002886	Left	3.87	-62.81	–66.68
ds002886	Right	3.87	–62.81	–66.68
ds003568	Left	–0.02	–81.09	–81.07
ds003568	Right	–0.02	-81.09	–81.07
ds001748	Right	–0.64	–90.20	–89.56
ds001748	Left	–0.64	–90.20	–89.56
ds003499	Left	–1.82	–157.03	–155.21
ds003499	Right	–1.82	–157.03	–155.21

It is important to note that our motion estimator works particularly well on the RUBIC site of HBN. This is explainable as it is an unseen site from the same base dataset as the training data.

**Figure 8:**
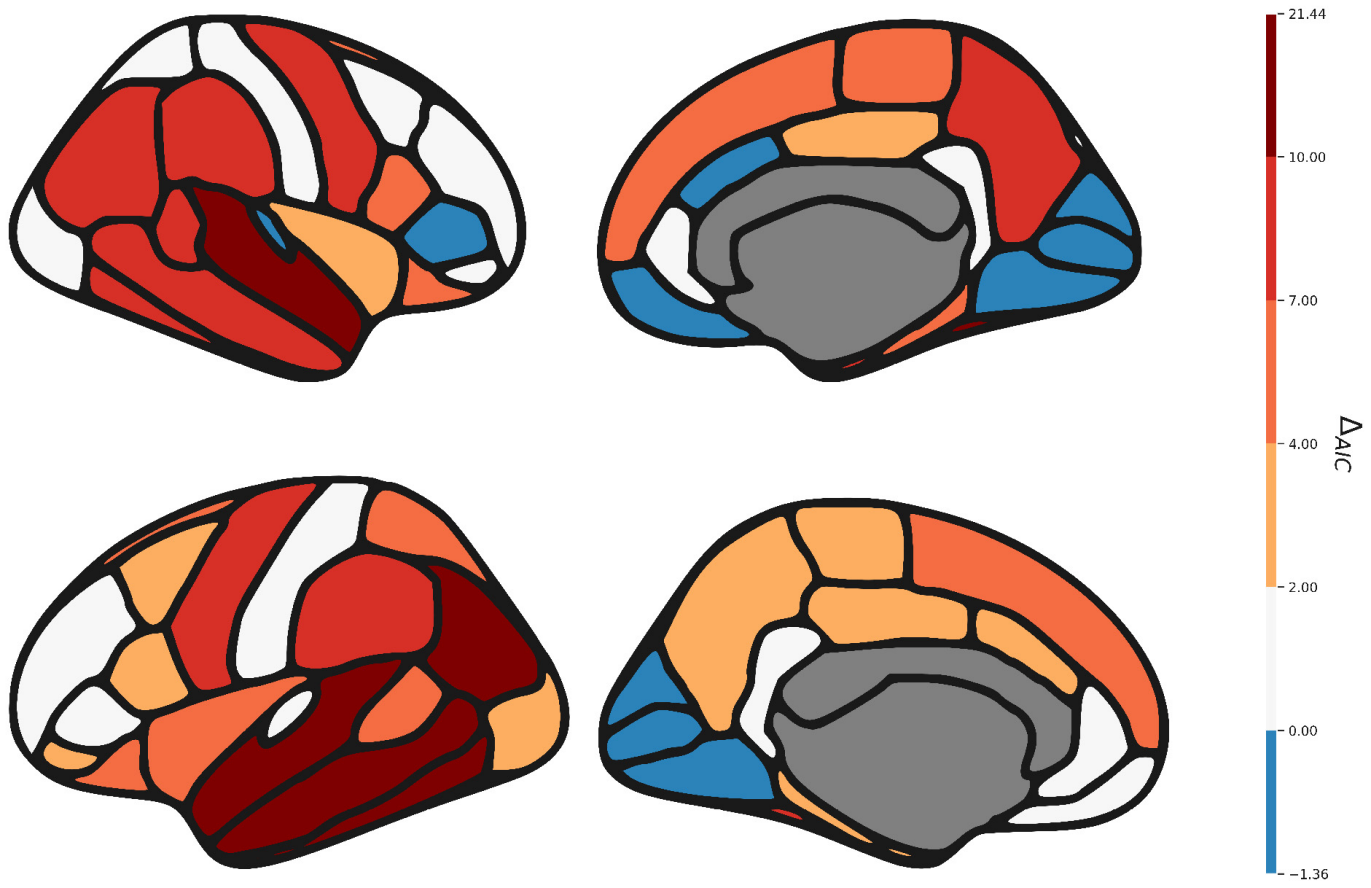
Median delta *AIC* (*AIC*_*base*_−*AIC*_*motion*_) for the thickness of each APARC regions. Higher means that the model using motion fits the data better.

In general, using the motion parameter improves the fitness of the model (see Figure 8). In particular, we can see that Middletemporal and Superiortemporal thickness, two measurements that we have identified as being highly sensitive to motion, are also the ones that benefit the most from introducing a motion parameter.

We also computed these values for area and volume (Supplementary Section 2.3). Expectedly, the measurement of area does not benefit from the introduction of the motion variable whereas it often improve the model for the volume.

## 4 Discussion

In this work, we leverage synthetic motion augmentation to generate a large labeled dataset using objective quantification based on simulated patient motion. We show that training our SFCN2 model on this synthetic dataset yields a high test *R*^2^, demonstrating the ability of our architecture to learn synthetic motion. Our model's correlation with MR-ART's labels also indicates good prediction of real motion without fine-tuning on real artifacts.

We then investigate the ability of our model to estimate known biases. First, we study how synthetic motion impacts the automatic measurement of cortical thickness and found a highly significant negative correlation, which is expected for real motion. This confirms that synthetic motion simulates not only direct artifacts but also other motion-related effects such as cortical thickness biases. To evaluate the relationship between cortical thickness and motion in real data, we visually show a clear relationship in HBN and MR-ART, while results are more uncertain for HCP-YA and HCP-EP. We explain this by the high quality standards of the two HCP studies, which are reflected in the overall motion distribution across the four datasets. This supports the adequacy of our model, as it displays distributions that match prior knowledge about data quality.

Furthermore, we test our best model on 15 independent separate datasets and obtain significant correlations between mean left hemisphere cortical thickness and predicted motion scores for 12 of them. When analyzing finer brain structures, we always find at least one region significantly correlated with our model's predictions. While it is known that motion affects cortical thickness differently depending on the structure, and even though we corrected our findings for multiple comparisons—it is likely that some of these findings are incidental. For example, we find only one affected structure out of 35 in dataset ds003499.

In addition, we test the relationship between age and motion, which has also been demonstrated in the literature, and found similar results. We observe significantly higher amounts of motion in both younger and older subjects.

As our analysis is primarily motivated by validating our motion quantifier, we focused on linear relationship for their simplicity. However, the data seem to display non linear tendencies that our GLM might not be able to represent.

After validating our model, we use it to study the impact of motion on the thickness of each region of the APARC atlas. We find that 35 out of 68 regions where significantly correlated in more than 50% of datasets. We identify the Middletemporal and Superiortemporal regions as the most frequently correlated with motion, regardless of the hemisphere. We then extend this frequency analysis to two other measurement, volume and area. We find that while volume showed similar patterns to thickness, area seems to be more robust to motion. Indeed, no region reported a frequency of correlation between motion and area higher than 50%.

Finally, we test the impact of our estimator on statistical model quality. We find that, in most cases, our estimator either improved or did not negatively impact model quality. The motion parameters was not beneficial when modeling the area, which is to be expected as area was less often correlated with motion. These results show the importance of our motion estimator to improve the quality of the statistical models in neuroscience, especially when studying cortical morphometry (Blumenthal et al., 2002; Reuter et al., 2015; Alexander-Bloch et al., 2016) on young subjects and population more prone to motion (Pardoe et al., 2016). To maximize the potential impact of our research, our model is made publicly and freely available on Zenodo and can be readily used through a tool called “Agitation3 as a CLI, a python library, a Boutiques container and a Nipoppy pipeline element.

## 5 Conclusion

This is the first attempt at correcting motion-related biases in automated anatomical measurements using a deep learning estimation of real patient motion through synthetic data. Our model is robust to variation in MRI hardware and software by comparing it to 15 datasets, unrelated to our training data. We also obtain good correlation with manually labeled motion scores on MR-ART. A relationship between age and motion is similarly studied, and our results are in agreement with previous research. We conclude that our model learns, from purely synthetic motion artifacts, a regression that can be readily applied to MRI studies using sequences close to MPRAGE, without prospective motion correction. This allows motion to be included as a variable in statistical analyses of population studies. We provide empirical evidence that our motion estimation is beneficial when fitting statistical models on metrics that are affected by motion such as thickness and volume. Including motion is important, especially for groups that tend to move more during scans, as motion can bias anatomical measurements. Our model provides a simple and reliable summary scalar that can support such analyses. To encourage the use of such a metric, we made both our model weight and a tool called “Agitation” publicly and freely available. “Agitation" can be used as a standalone CLI, a python library, a Boutiques container and a Nipoppy pipeline element to ease the adoption of the tool by the neuroscience community.

Future research should focus on testing this method on different scanners and studying how it could be adapted for volumes using prospective motion correction. It would also be interesting to expand this method to other kinds of artifacts that can be simulated, to design more accurate artifact simulators, and to use a broader set of regression metrics. Finally, future work using the motion quantification model should look into non linear models.

## Data Availability

Publicly available datasets were analyzed in this study. This data can be found at: https://fcon_1000.projects.nitrc.org/indi/cmi_healthy_brain_network/MRI_EEG.html, https://www.humanconnectome.org/study/hcp-young-adult/overview, https://www.humanconnectome.org/study/human-connectome-project-for-early-psychosis, https://doi.org/10.18112/openneuro.ds004173.v1.0.2, https://openneuro.org/datasets/ds000115/versions/00001, https://openneuro.org/datasets/ds000144/versions/00002, https://openneuro.org/datasets/ds000256/versions/00002, https://doi.org/10.18112/openneuro.ds001486.v1.3.1, https://doi.org/10.18112/openneuro.ds001748.v1.0.4, https://doi.org/10.18112/openneuro.ds002424.v1.2.0, https://github.com/OpenNeuroDatasets-JSONLD/ds002862, https://doi.org/10.18112/openneuro.ds002886.v1.1.0, https://doi.org/10.18112/openneuro.ds003499.v1.0.1, https://doi.org/10.18112/openneuro.ds003568.v1.0.4, and https://doi.org/10.18112/openneuro.ds005234.v2.1.7.
