# Supplementary material for: Estimation of head motion in structural MRI and its impact on cortical morphometry
Source: Front Neurosci. 2026 May 8;20:1817743. doi: 10.3389/fnins.2026.1817743 (PMC13194591; doi:10.3389/fnins.2026.1817743)
Supplement: Supplementary file 1 [file Data_Sheet_1.pdf]

Supplementary Material

1 METHOD

1.1 Motion Generation

We used the `RandomMotion` transform from TorchIO (Pérez-García et al., 2021), it implements the procedure first described by Shaw et al. (2019). Given a chosen number of motion events n, the transform constructs a normalised time axis $t \in [0, 1]$ and places n events by dividing the axis into $n + 1$ equal intervals of step size $\Delta t = \frac{1}{n+1}$. Uniform noise is added to each event time as

$$t_i = i \cdot \Delta t + \eta_i, \quad \eta_i \sim \mathcal{U}(-\epsilon \cdot \Delta t,\ \epsilon \cdot \Delta t)\,, \quad i = 1, \dots, n, \tag{S1}$$

where $\epsilon = 0.3$ is a perturbation factor. Independently, rotation and translation parameters

$$\boldsymbol{R}_i = (R_X, R_Y, R_Z)_i \sim \mathcal{U}(R_{\min}, R_{\max})^3, \qquad \boldsymbol{T}_i = (T_X, T_Y, T_Z)_i \sim \mathcal{U}(T_{\min}, T_{\max})^3 \tag{S2}$$

are sampled for each event $i = 1, \dots, n$. Each rigid transform $\mathcal{T}_i(\boldsymbol{R}_i, \boldsymbol{T}_i)$ is applied to the original volume V_0, yielding $n+1$ volumes $\{V_0, V_1, \dots, V_n\}$. Their respective k-spaces are obtained via the discrete Fourier transform, $S_i = \mathcal{F}(V_i)$.

To assemble the corrupted k-space, the last spatial axis (of length L) is used as the simulated phase-encoding direction and partitioned into $n + 1$ non-overlapping contiguous segments

and the final corrupted volume is recovered by the inverse Fourier transform, $V^* = \mathcal{F}^{-1}(S^*)$. Figure S1 illustrates the sampled rotation, translation, and event parameters for a single realisation with $n = 4$.

In our study, the number of motion events n is sampled uniformly from the integers $\{2, \dots, 8\}$. To obtain an approximately uniform distribution of motion severities, we first sample a target motion score m_t from $\mathcal{U}(0.01, 4)$. We then generate candidate motion transforms and retain the first candidate whose resulting motion score m satisfies $|m - m_t| < \epsilon$, with $\epsilon = 0.02$. This rejection-sampling procedure allows us to match the simulated transform to a desired motion level.

For each candidate transform, we adapt the rotation and translation sampling ranges to the target score m_t. Specifically, we draw symmetric bounds as

$$\begin{aligned} R_{\max} &\sim \mathcal{U}(0, 2m_t), \qquad & R_{\min} &= -R_{\max}, \\ T_{\max} &\sim \mathcal{U}(0, m_t), \qquad & T_{\min} &= -T_{\max}. \end{aligned}$$

The event-wise rotation and translation parameters are then sampled uniformly within these ranges. This heuristic increases the likelihood of obtaining a transform with a motion score close to the desired target without relying on excessively large parameter ranges.

1.2 Motion Score Examples

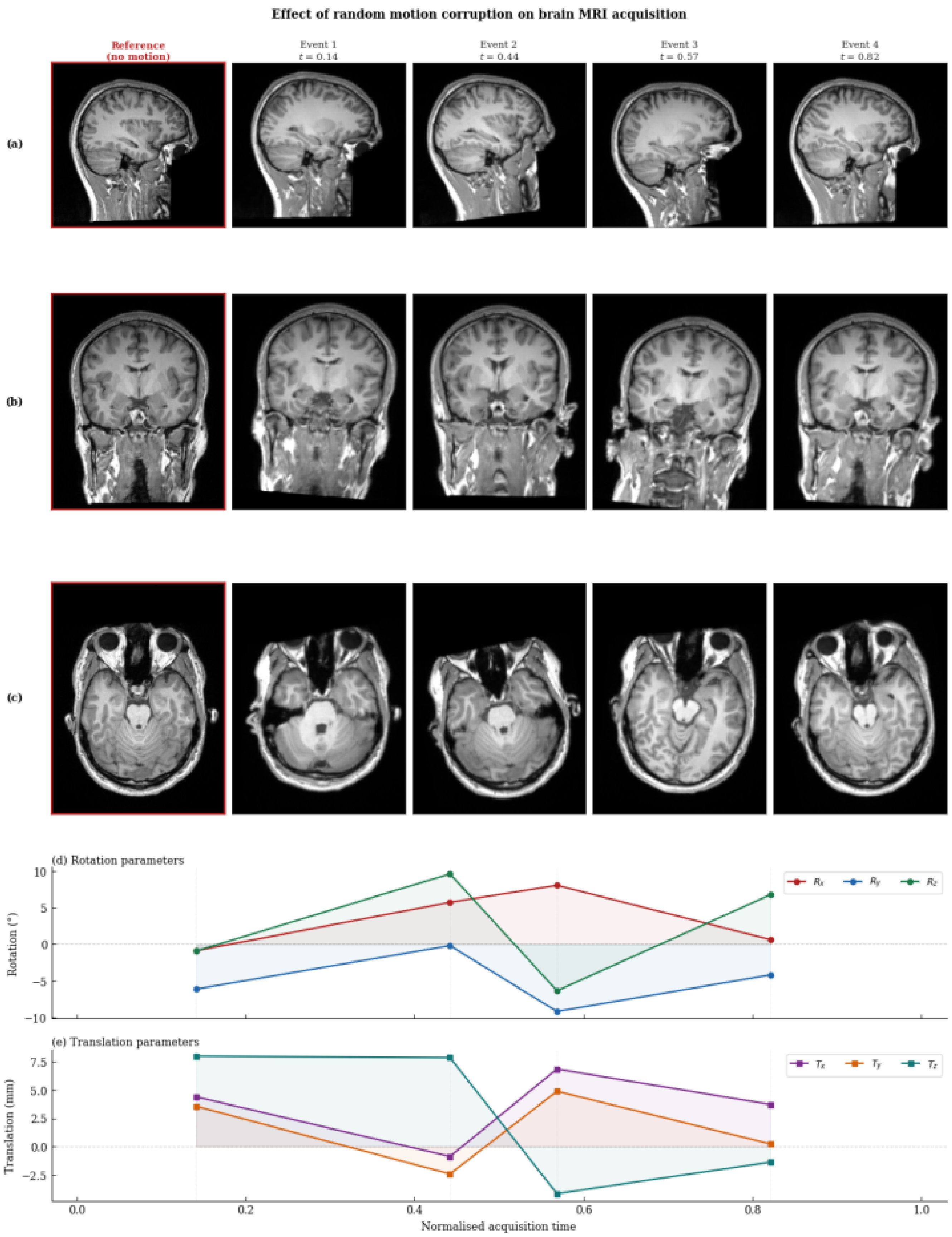

Figure S1. Visualisation of motion generation. (a),(b) and (c) present slices of the brain at each given transformation. (d) and (e) show the sampled rotations and translation for each time step.

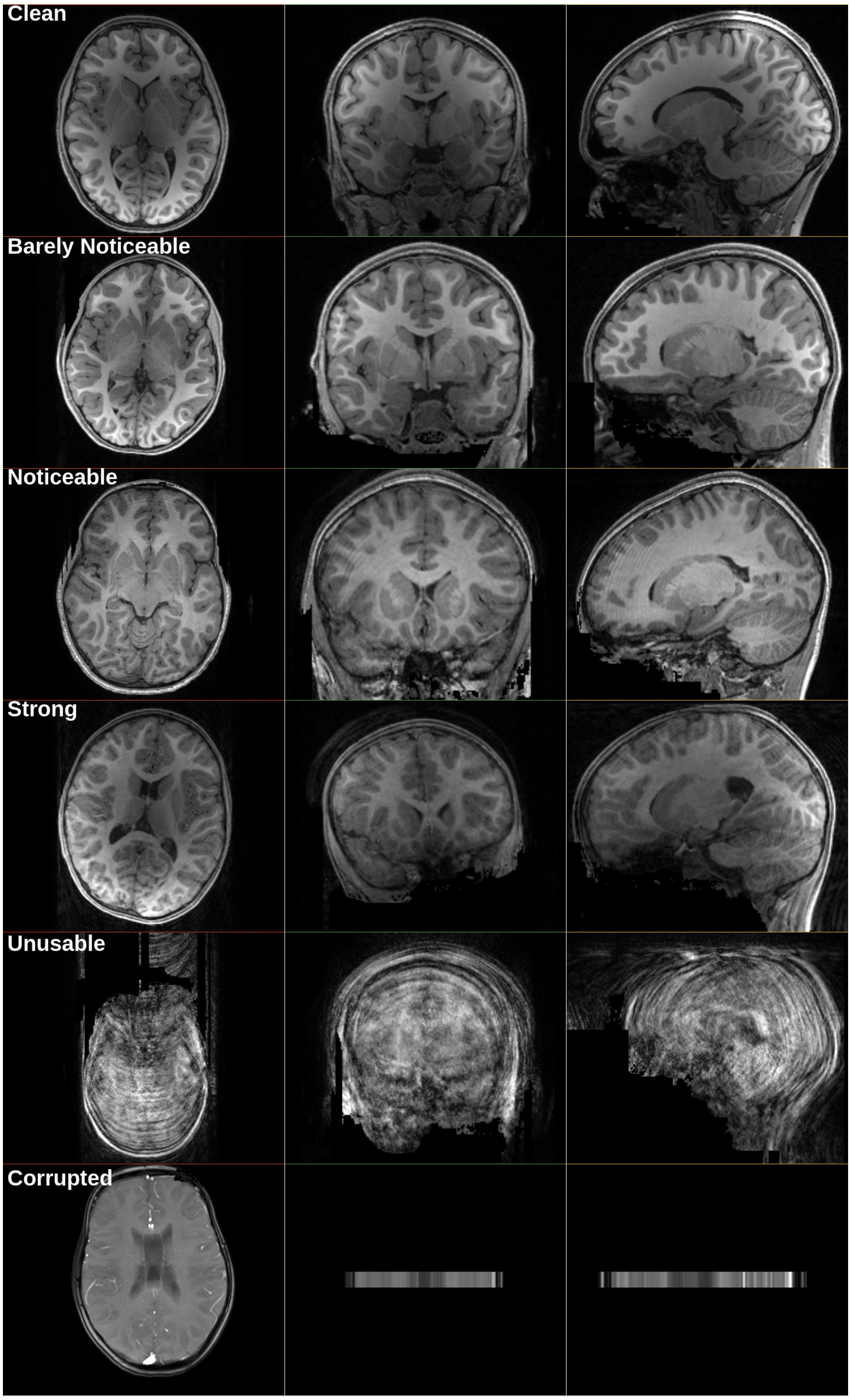

Figure S2. Examples from Healthy Brain Network (HBN) presented to Rater Two and Three

2 IMPACT OF MOTION ON CORTICAL MORPHOMETRY

2.1 Correlation between Cortical Thickness and Motion for each Structure

Hemisphere	Structure	Percentage of Significant Correlations
Right	Middletemporal	80.00%
Right	Superiortemporal	80.00%
Right	Fusiform	80.00%
Left	Middletemporal	80.00%
Right	Bankssts	73.33%
Left	Superiortemporal	73.33%
Left	Supramarginal	73.33%
Left	Temporalpole	73.33%
Right	Inferiorparietal	73.33%
Right	Inferiortemporal	73.33%
Left	Precentral	73.33%
Left	Fusiform	73.33%
Right	Supramarginal	73.33%
Right	Parahippocampal	66.67%
Left	Inferiortemporal	66.67%
Left	Inferiorparietal	66.67%
Right	Temporalpole	66.67%
Left	Lateralorbitofrontal	66.67%
Left	Superiorfrontal	60.00%
Right	Paracentral	60.00%
Right	Parsopercularis	60.00%
Right	Precentral	60.00%
Right	Lateralorbitofrontal	60.00%
Right	Entorhinal	60.00%
Right	Superiorfrontal	60.00%
Left	Bankssts	60.00%
Left	Insula	60.00%
Left	Entorhinal	53.33%
Right	Insula	53.33%
Left	Lateraloccipital	53.33%
Right	Precuneus	53.33%
Left	Caudalanteriorcingulate	53.33%
Left	Superiorparietal	53.33%
Left	Precuneus	53.33%
Left	Parahippocampal	53.33%
Left	Caudalmiddlefrontal	46.67%
Right	Postcentral	46.67%
Left	Paracentral	46.67%

Continued on next page

Hemisphere	Structure	Percentage of Significant Correlations
Left	Isthmuscingulate	46.67%
Right	Caudalmiddlefrontal	46.67%
Left	Posteriorcingulate	46.67%
Left	Parsopercularis	46.67%
Right	Posteriorcingulate	40.00%
Left	Parsorbitalis	40.00%
Right	Pericalcarine	40.00%
Right	Superiorparietal	40.00%
Left	Postcentral	40.00%
Left	Rostralmiddlefrontal	40.00%
Right	Parstriangularis	33.33%
Right	Rostralmiddlefrontal	33.33%
Left	Parstriangularis	33.33%
Left	Cuneus	33.33%
Left	Transversetemporal	33.33%
Left	Frontalpole	26.67%
Left	Rostralanteriorcingulate	26.67%
Right	Transversetemporal	26.67%
Right	Parsorbitalis	26.67%
Left	Medialorbitofrontal	26.67%
Right	Lateraloccipital	26.67%
Left	Lingual	20.00%
Right	Rostralanteriorcingulate	20.00%
Right	Isthmuscingulate	20.00%
Right	Frontalpole	20.00%
Right	Cuneus	20.00%
Right	Caudalanteriorcingulate	20.00%
Left	Pericalcarine	20.00%
Right	Medialorbitofrontal	13.33%
Right	Lingual	13.33%

Table S1: Frequency analysis of the correlation between motion and thickness for all the APARC substructure on both hemisphere

2.2 Study of Δ_{AIC} for each Structure

Structure	Hemisphere	Δ_{AIC}
Lateralorbitofrontal	Left	11.22
Middletemporal	Right	11.07

Continued on next page

Structure	Hemisphere	Δ_{AIC}
Middletemporal	Left	8.43
Superiortemporal	Right	7.50
Superiortemporal	Left	7.36
Lateralorbitofrontal	Right	7.10
Superiorfrontal	Left	7.02
Fusiform	Left	6.77
Precuneus	Left	6.41
Inferiortemporal	Left	6.00
Precentral	Left	5.83
Precentral	Right	5.68
Rostralmiddlefrontal	Left	5.49
Caudalmiddlefrontal	Left	5.27
Supramarginal	Left	5.21
Rostralanteriorcingulate	Right	4.96
Superiorfrontal	Right	4.78
Inferiortemporal	Right	4.75
Precuneus	Right	4.70
Temporalpole	Right	4.54
Temporalpole	Left	4.25
Medialorbitofrontal	Right	3.78
Bankssts	Right	3.53
Parsorbitalis	Right	3.48
Fusiform	Right	3.45
Parsorbitalis	Left	3.39
Posteriorcingulate	Left	3.25
Parsopercularis	Right	3.22
Superiorparietal	Right	3.02
Rostralmiddlefrontal	Right	2.91
Inferiorparietal	Right	2.91
Caudalanteriorcingulate	Right	2.85
Paracentral	Right	2.80
Entorhinal	Left	2.62
Rostralanteriorcingulate	Left	2.37
Supramarginal	Right	2.22
Parstriangularis	Left	2.12
Superiorparietal	Left	1.99
Frontalpole	Right	1.95
Parahippocampal	Right	1.84
Entorhinal	Right	1.74
Bankssts	Left	1.65
Inferiorparietal	Left	1.48
Posteriorcingulate	Right	1.07

Continued on next page

Structure	Hemisphere	Δ_{AIC}
Insula	Left	1.05
Caudalanteriorcingulate	Left	1.01
Medialorbitofrontal	Left	1.01
Caudalmiddlefrontal	Right	0.67
Insula	Right	0.66
Paracentral	Left	0.64
Lateraloccipital	Right	0.53
Cuneus	Left	0.40
Postcentral	Right	0.34
Parahippocampal	Left	0.31
Cuneus	Right	0.24
Parstriangularis	Right	0.19
Parsopercularis	Left	0.06
Lingual	Left	0.04
Postcentral	Left	-0.36
Lateraloccipital	Left	-0.38
Isthmuscingulate	Left	-0.41
Frontalpole	Left	-0.41
Pericalcarine	Right	-0.75
Pericalcarine	Left	-0.79
Lingual	Right	-0.98
Isthmuscingulate	Right	-1.26
Transversetemporal	Left	-1.44
Transversetemporal	Right	-1.54

Table S2: AIC comparison on the impact of introducing a motion parameter when modelling the cortical thickness of each APARC structure for each hemisphere

2.2.1 Frequency Analysis Plots on Area and Volume

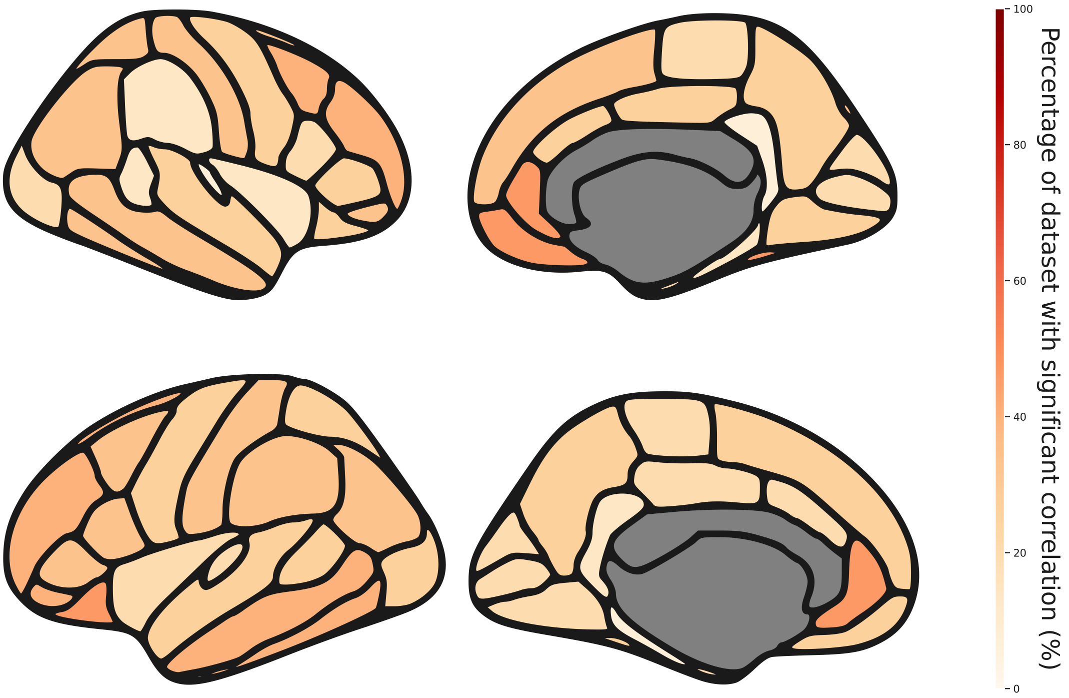

Figure S3. Percentage of dataset with a significant correlation between area and motion for each structure

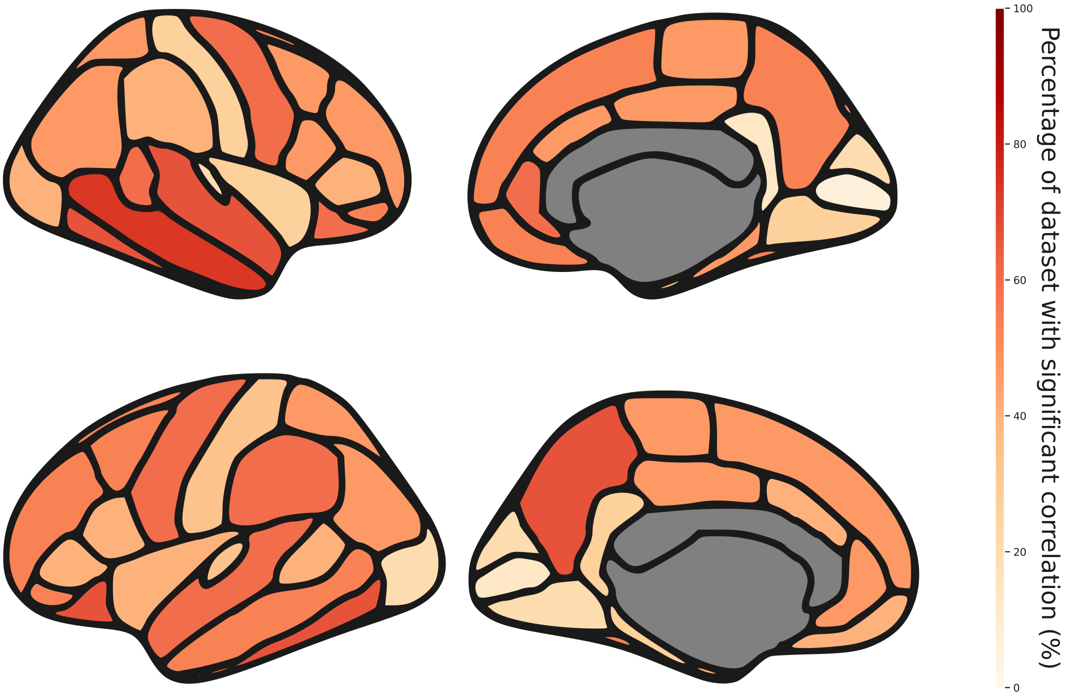

Figure S4. Percentage of dataset with a significant correlation between volume and motion for each structure

2.3 Δ_{AIC} on Volume and Area for each Structure

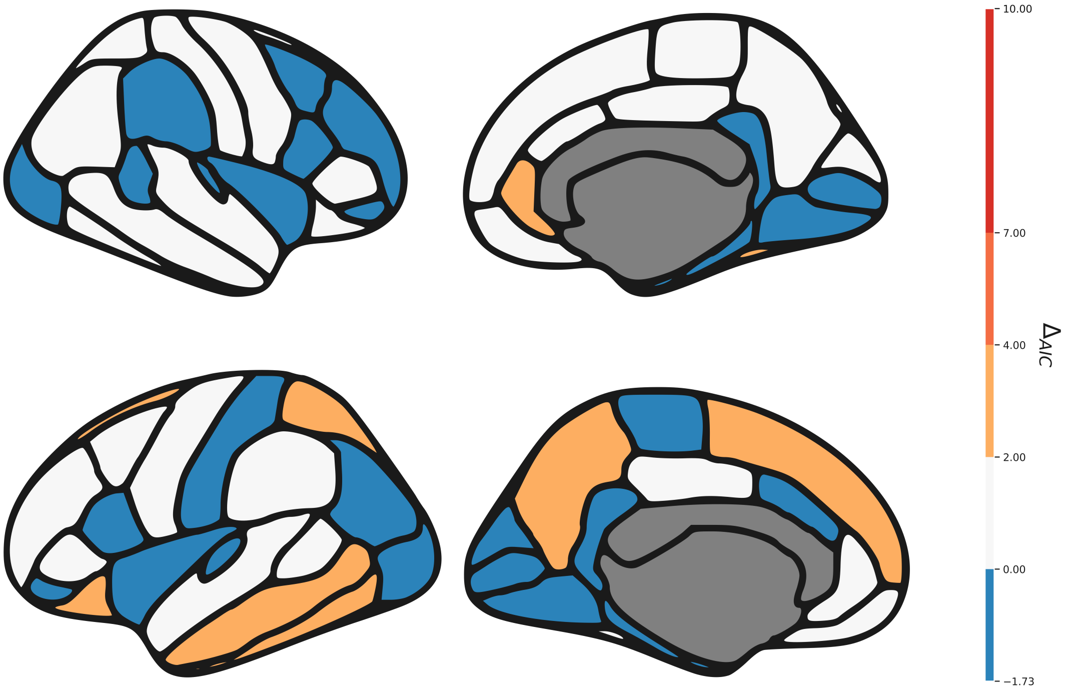

Figure S5. Median Delta AIC ($AIC_{base} - AIC_{motion}$) for the area of each APARC regions.

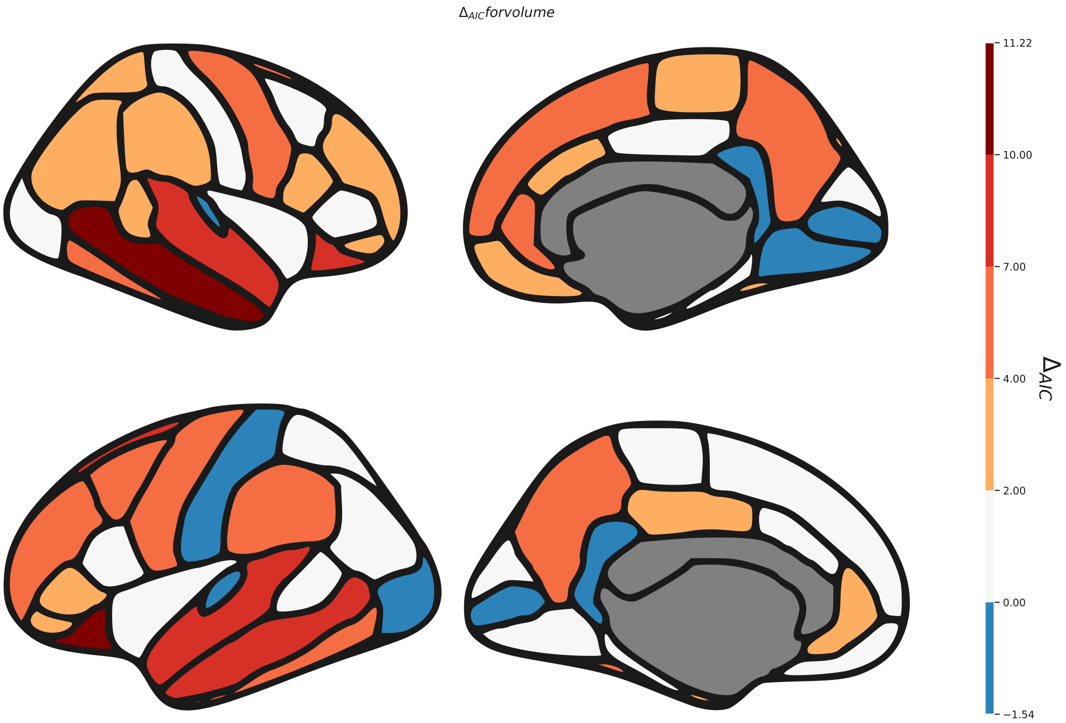

Figure S6. Median Delta AIC ($AIC_{base} - AIC_{motion}$) for the volume of each APARC regions.

REFERENCES

Pérez-García, F., Sparks, R., and Ourselin, S. (2021). TorchIO: A Python library for efficient loading, preprocessing, augmentation and patch-based sampling of medical images in deep learning. *Computer Methods and Programs in Biomedicine* 208, 106236. doi:10.1016/j.cmpb.2021.106236

Shaw, R., Sudre, C., Ourselin, S., and Cardoso, M. J. (2019). MRI k-Space Motion Artefact Augmentation: Model Robustness and Task-Specific Uncertainty. In *Proceedings of The 2nd International Conference on Medical Imaging with Deep Learning* (PMLR), 427–436. ISSN: 2640-3498
